# Continuous intra-articular infusion anesthesia for pain control after total knee arthroplasty: study protocol for a randomized controlled trial

**DOI:** 10.1186/1745-6215-15-245

**Published:** 2014-06-23

**Authors:** Da Guo, Xue-Wei Cao, Jin-Wen Liu, Wen-Wei Ouyang, Jian-Ke Pan, Jun Liu

**Affiliations:** 1 Department of Orthopedic Surgery, The Second School of Clinical Medicine, Guangzhou University of Chinese Medicine, No. 111 Dade Road, Guangzhou, Guangdong 510120, China; 2 The Clinical Epidemiology Application Laboratory, The Second School of Clinical Medicine, Guangzhou University of Chinese Medicine, No. 111 Dade Road, Guangzhou, Guangdong 510120, China

**Keywords:** Total knee arthroplasty, Postoperative pain control, Continuous intra-articular infusion anesthesia, Randomized controlled trial

## Abstract

**Background:**

Postoperative pain control after total knee arthroplasty (TKA) remains a great challenge. The management of pain in the immediate postoperative period is one of the most critical aspects to allow speedier rehabilitation and reduce the risk of postoperative complications. Recently, periarticular infiltration anesthesia has become popular, but the outcome is controversial. Some studies have shown transient effects, “rebound pain”, or no effectiveness in pain control. Continuous intra-articular infusion technique has been introduced to improve these transient effects, but more clinical studies are needed. Furthermore, the potential risk of early periprosthetic joint infection is causing concerning. We plan to compare continuous intra-articular infusion anesthesia with epidural infusion anesthesia after TKA to assess the effectiveness of this technique in reducing pain, in improving postoperative function, and to look at the evidence for risk of early infection.

**Methods/design:**

This trial is a randomized, controlled study. Patients (*n* = 214) will be randomized into two groups: to receive continuous intra-articular infusion anesthesia (group C); and epidural infusion anesthesia (group E). For the first 3 postoperative days, pain at rest, active range of motion (A-ROM), rescue analgesia and side effects will be recorded. At 3-month and 6-month follow-up, A-ROM, C-reactive protein, erythrocyte sedimentation rate, and synovial fluid cell count and culture will be analyzed.

**Discussion:**

The results from this study will provide clinical evidence on the efficacy of a continuous intra-articular infusion technique in reducing pain, postoperative functional improvement and safety. It will be the first randomized controlled trial to investigate infection risk with local anesthesia after TKA.

**Trial registration:**

ClinicalTrials.gov identifier: ChiCTR-TRC-13003999

## Background

Pain after total knee arthroplasty (TKA) is usually severe. It causes a state of discomfort that may directly influence patients' functional recovery. The pain involved has specific characteristics: a 55 to 60% incidence at rest and up to70% upon mobilization; high or very high intensity; and pain peaking at 3 to 6 hours after surgery and continuing for the following 72 hours [1,2]. Anesthetic techniques such as epidural analgesia or peripheral nerve block may reach effective analgesic levels [3-5] in combination with opioid analgesics and non-steroidal anti-inflammatory drugs. Both techniques either have significant demands on technical skills, have potential side effects, or are costly [6-8]. Recently, local anesthesia techniques have become popular, including: (1) periarticular infiltration with or without local intermittent administration of anesthetics with an intra-articular catheter [6,7,9-12]; and (2) a combined technique with infiltration plus continuous infusion [8,13,14]. Local anesthesia has shown several advantages (less intensity of postoperative pain, less consumption of rescue analgesics and earlier discharge) when compared to other regional or purely systemic approaches [15-17].

There is some controversy surrounding periarticular infiltration anesthesia. Some studies, using single-dose or combined-dose periarticular infiltration, have shown transient effects, “rebound pain”, or no effectiveness in pain control [12,18-21]. The continuous intra-articular infusion technique has been introduced to improve these transient effects. Two previous studies [8,14] using continuous intra-articular infusion anesthesia showed better pain relief and less opioid use than a control group using saline infusion. Further investigation is needed to assess the efficacy of continuous intra-articular infusion anesthesia. One of the most concerning issues is whether continuous intra-articular infusion would increase the risk of early periprosthetic joint infection (PJI). Early diagnosis of PJI is challenging; at the present time, diagnosis of PJI remains dependent on clinical judgment and reliance on standard clinical tests including serologic tests, analysis of aspirated joint fluid, and interpretation of intraoperative tissue and fluid test results.

For this purpose, the aims of our study were: (1) to analyze the efficacy of continuous intra-articular infusion anesthesia in postoperative pain control and functional recovery compared with epidural analgesia; and (2) to investigate PJI markers, such as erythrocyte sedimentation rate (ESR), C-reactive protein (CRP), synovial fluid cell count and culture during follow-up, to determine whether the risk of early PJI increases with a continuous intra-articular infusion procedure.

## Methods/design

### Study design

This is a randomized, controlled prospective study. The trial protocol has been approved by the Research Ethical Committee of Guangdong Provincial Hospital of Traditional Chinese Medicine (TCM) (reference, B2011-38-01). The trial will be conducted in accordance with the Helsinki Declaration, and will be monitored by the trial agency at Guangdong Provincial Hospital of TCM.

### Recruitment and consent

**Figure 1:**
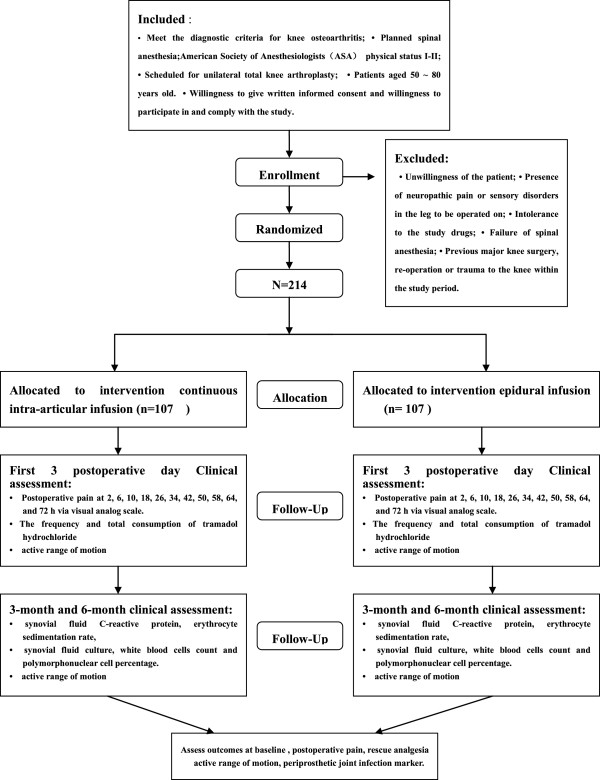
Flow-chart of the study.

Patients scheduled for primary TKA at the Department of Orthopedic Surgery, Guang Dong Provincial Hospital of TCM, will be recruited, with a target sample size of 214 subjects. All candidates will go through a standardized interview process and receive more information about the study and the treatments. The participants’ written consents will be obtained. The purpose and procedures, and potential risks and benefits, of the study will also be explained thoroughly to the participants. Participants are able to withdraw from the study at any time without consequence. The trial will be executed from February 2014 to December 2015 including enrollment and follow-up (Figure 1).

### Inclusion criteria

Participants meeting the following criteria will be included:

• Meet the diagnostic criteria for knee osteoarthritis;

• Planned spinal anesthesia; American Society of Anesthesiologists (ASA) physical status I-II;

• Scheduled for unilateral TKA;

• Patients aged 50 to 80 years old.

• Willingness to give written informed consent and willingness to participate in and comply with the study.

### Exclusion criteria

Participants meeting one or more of the following criteria will be excluded:

• Unwillingness of the patient;

• Presence of neuropathic pain or sensory disorders in the leg to be operated on;

• Intolerance to the study drugs;

• Failure of spinal anesthesia;

• Previous major knee surgery, re-operation or trauma to the knee within the study period.

### Interventions

All recruited patients will receive combined spinal and epidural anesthesia at the L3-4 level. For all the patients, cefamandole (2 g) will be administered intravenously 30 minutes before and after surgery. Low-molecular-weight heparin (4,500 U) for thromboprophylaxis will be administered subcutaneously 8 hours postoperatively and then once daily until week 2. In group E, the heparin administration will be delayed until epidural catheter withdrawal on postoperative day (POD) 2. A standardized intraoperative regimen for fluid administration will be applied which consists of 0.9% saline at 5 ml/kg per hour and colloid (Voluven; Fresenius Kabi, Bad Homburg, Germany) at 7.5 ml/kg per hour.

For multi-model pain management, preoperative oral doses of 400 mg celecoxib daily will be administered from day 2 before surgery. Postoperatively, all patients will receive 40 mg of parecoxib sodium twice daily intravenously until POD 3, followed by oral doses of 200 mg celecoxib every 12 hours until discharge. Tramadol hydrochloride injection will be applied for rescue analgesia.

The same surgeon will perform all operations. A Gemini MK II prosthesis (LINK, Hamburg, Germany) will be inserted via a standard medial parapatellar approach in all patients.

#### ***Cocktail anesthetic preparation***

Cocktail anesthetic will be prepared for continuous intra-articular infusion, containing 150 mg ropivacaine (20 ml), 3 mg morphine sulfate (0.3 ml), 30 mg ketorolac tromethamine (1 ml), 0.5 μg 1:1000 epinephrine (0.5 ml), mixed with normal saline into a total volume of 150 ml.

#### ***Continuous intra-articular infusion anesthesia group (group C)***

In group C, 50 ml of cocktail anesthetic will be applied via periarticular infiltration before insertion of the prosthesis. A multi-holed catheter will be placed into the lateral parapatellar gutter. The remaining 100 ml will be used as continuous intra-articular infusion with a patient-controlled analgesic (PCA) pump (Fornia, Zhuhai, China) at a speed of 2 ml/hour. After the wound is sutured, the catheter will be connected to the PCA pump which is activated after surgery and removed 48 hours later. The PCA pump will be set to deliver a 0.5-ml dose with a 15-minute lockout interval.

#### ***Epidural infusion anesthesia group (group E)***

In group E, the epidural catheter position will be checked with a test dose of 3 ml lidocaine-adrenalin (20 mg/ml and 5 μg/ml), followed by a bolus of 7 ml ropivacaine (2 mg/ml). A PCA pump containing 200 mg of ropivacaine (2 mg/ml) and 4 mg of morphine (0.04 mg/ml) will be connected with a flow rate of 2 ml/hour for 48 hours postoperatively. The PCA will be programmed to provide a 0.5-ml dose with a lockout interval of 15 minutes.

### Randomization and blinding

Random assignment was performed before surgery by using a computer generated, blocked random-allocation sequence with a 1:1 ratio. This study cannot be blinded because the different catheter placement is obvious.

### Primary outcome measures

#### ***Visual analogue scale***

The visual analog scale (VAS) has been used extensively for rating pain intensity in previous studies [22-24]. Postoperative pain at 2, 6, 10, 18, 26, 34, 42, 50, 58, 64, and 72 hours after surgery will be recorded using a VAS. The VAS consists of a 10-cm horizontal line with 1-cm vertical lines at each end labeled "no pain" (left side) and "worst possible pain" (right side).

#### ***Rescue analgesia frequency and consumption***

Usually the need for supplementary treatment with systemic opioids is a reliable indicator when concerned about postoperative pain control. In previous studies, it has been argued that the use of strong or long-term rescue analgesia in both treatment groups may have reduced pain too much and “washed out” any differences between the two treatments. In this study, we use tramadol hydrochloride injection, which is a mid-intensity analgesic that will facilitate observing the differences in pain control between groups. The frequency and total consumption during the first 3 postoperative days will be recorded.

#### ***Active range of motion***

Traditionally, range of motion has been used as an outcome measure after TKA to evaluate functional recovery and the success of the type of analgesia used [4-6,12]. However, we think active range of motion (A-ROM) represents a more realistic criterion for assessment of functioning and analgesia efficiency after TKA. A-ROM will be measured in the first 3 postoperative days and at the 3-month and 6-month follow-up.

### Secondary outcome measures

#### ***Periprosthetic joint infection markers***

Early diagnosis of PJI is challenging; at the present time, diagnosis of PJI remains dependent on clinical judgment and reliance on standard clinical tests including serologic tests, analysis of aspirated joint fluid, and interpretation of intraoperative tissue and fluid test results. During 3-month and 6-month follow-up, the CRP, ESR, synovial fluid culture, white blood cell (WBC) count and polymorphonuclear cell percentage (%PMN) will be measured.

### Safety

The presence of side effects during the postoperative period will be recorded, including nausea, vomiting, constipation and other adverse events.

### Sample size

Calculation of sample size was based on two previous studies [20,25]. We assumed that postoperative pain during the 24- to 48-hour period will reach peak value and need effective pain control. Mean VAS scores over 24 to 48 hours for the two modes of analgesia were extracted. Group sample sizes of 93 for each group will achieve 80% power to detect a difference of 1.0 between the null hypothesis that both group means are 2.8 and the alternative hypothesis that the mean of group E is 1.8, with estimated group standard deviations of 3.1 and 1.4 and with a significance level (alpha) of 0.05 using a two-sided *t*-test. We plan to enroll a total of 214 participants with 107 in each group, allowing for a 15% withdrawal rate.

### Data analysis

The statistical analysis will be performed using SPSS (Version 17.0) software (SPSS Inc., Shanghai, China). Normally distributed continuous variables will be compared using Student’s *t*-test. VAS and A-ROM will be analyzed with repeated measures for general linear models and multivariate analysis of variance. Data will be expressed as means (standard deviation), absolute numbers, and frequencies, when appropriate. *P* < 0.05 will be considered statistically significant for all comparisons.

## Discussion

This study will provide clinical evidence on the efficacy of a continuous intra-articular infusion technique in reducing pain and improving postoperative function. In TKA, this remains a significant problem in the early postoperative period. Reduction of the patient's discomfort is the primary goal; reduction of the cost of narcotic use is a secondary goal. The epidural analgesia has been regarded as the gold standard in postoperative pain management, and it is associated with potential problems such as motor block, urinary retention and epidural bleeding (with anticoagulation therapy), and necessitates intensive observation of patients. The primary purpose of our study is to evaluate the efficacy of a continuous intra-articular infusion technique in relieving postoperative pain, reducing the need for postoperative narcotics, and improvement in postoperative function.

This study will be the first randomized controlled trial to investigate infection risk with a continuous intra-articular infusion technique after TKA. There has been concern about the risk of early PJI with periarticular multimodal drug injection and use of intra-articular catheters after TKA. Previous studies of local infiltration analgesia after unicompartmental knee arthroplasty or TKA have not found any increase in infection [7,15,20,26], but the evidence has been weak in most studies. At the present time, diagnosis of PJI remains dependent on clinical judgment and reliance on standard clinical tests including serologic tests and analysis of aspirated joint fluid. For this purpose, we will measure the ESR, CRP, synovial fluid cultures, WBC count, and %PMN at the 3-month and 6-month follow-up to identify early infections related to the surgical procedure.

## Trial status

Recruitment will commence in February 2014, and the trial is scheduled to end in December 2015.

## Abbreviations

A-ROM: active range of motion; CRP: C-reactive protein; ESR: erythrocyte sedimentation rate; PCA: patient-controlled analgesic; PJI: periprosthetic joint infection; %PMN: polymorphonuclear cell percentage; TCM: Traditional Chinese Medicine; TKA: total knee arthroplasty; VAS: visual analog scale; WBC: white blood cell; POD: postoperative day.

## Competing interests

The authors declare that they have no competing interests.

## Authors’ contributions

JL conceived the study and prepared the initial protocol, and was in charge of coordination. DG drafted the manuscript and participated in the study design. XWC participated in the protocol and performed surgery. WWO and JKP helped to develop the study analysis. JWL made amendments and participated in designing the trial protocol. All authors read and approved the final manuscript.
